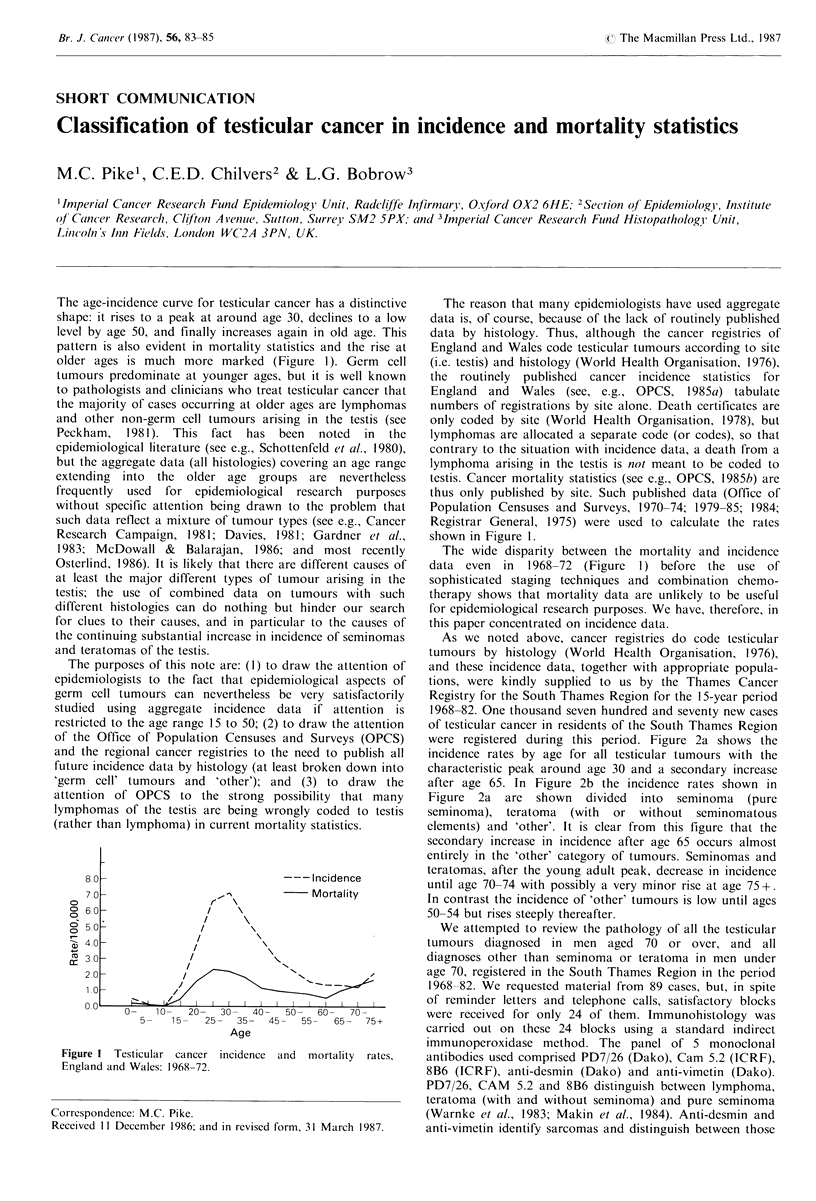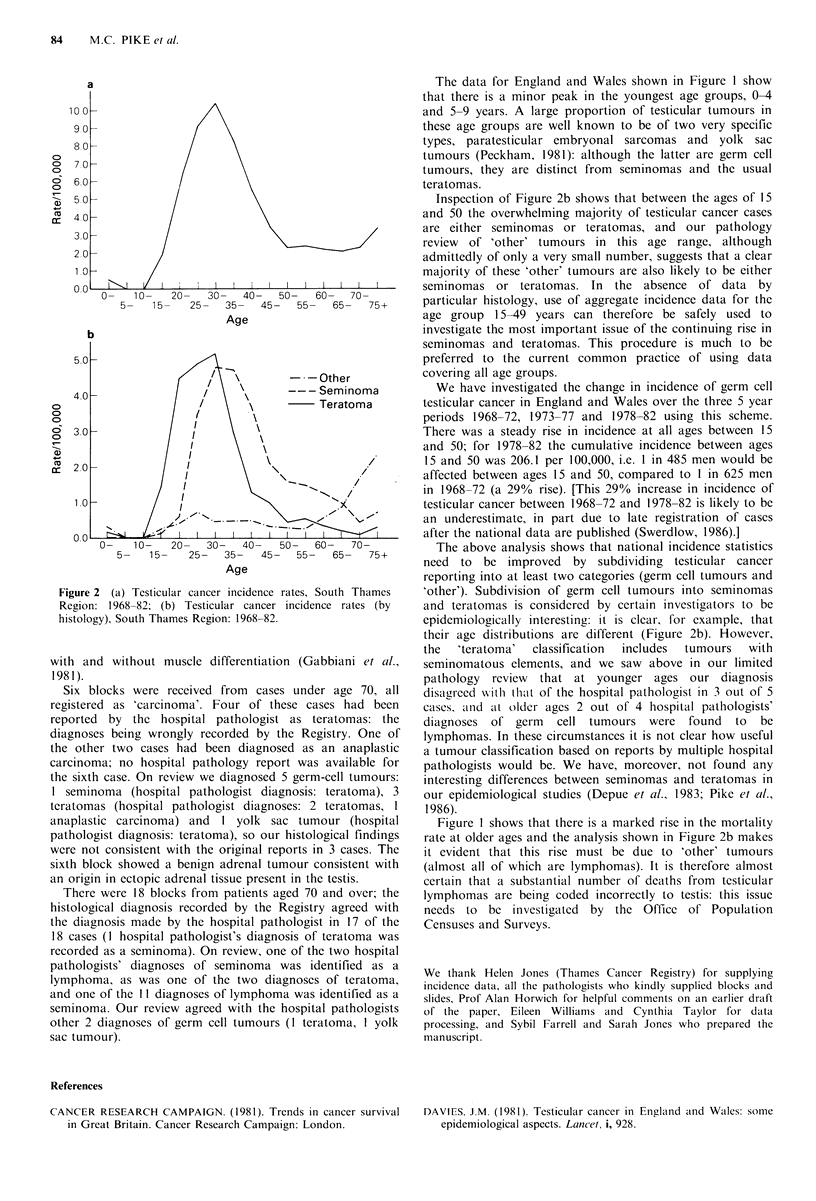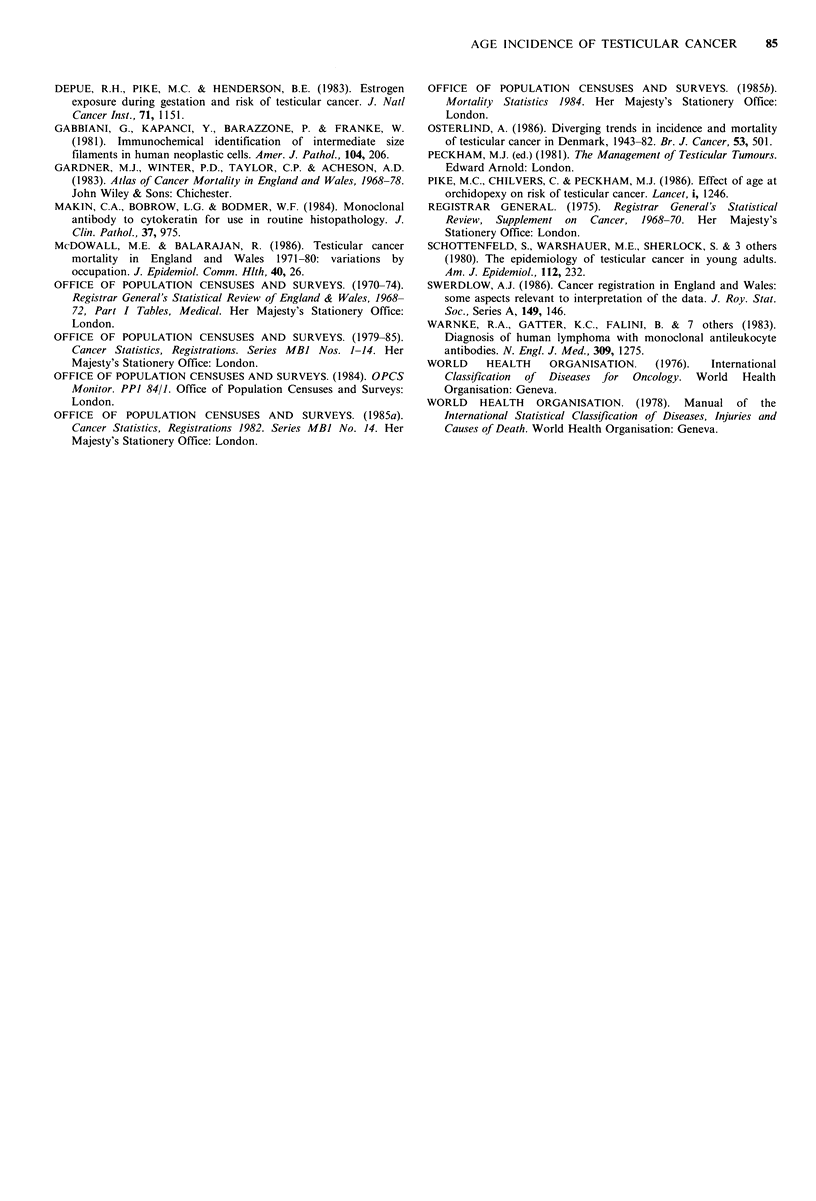# Classification of testicular cancer in incidence and mortality statistics.

**DOI:** 10.1038/bjc.1987.159

**Published:** 1987-07

**Authors:** M. C. Pike, C. E. Chilvers, L. G. Bobrow


*Br. J. Cancer* (1987), **56**, 83–85 © The Macmillan Press Ltd., 1987

**SHORT COMMUNICATION**

### Classification of testicular cancer in incidence and mortality statistics

M.C. Pike[1], C.E.D. Chilvers[2] & L.G. Bobrow[3]

[1]*Imperial Cancer Research Fund Epidemiology Unit, Radcliffe Infirmary, Oxford OX2 6HE;* [2]*Section of Epidemiology, Institute of Cancer Research, Clifton Avenue, Sutton, Surrey SM2 5PX; and* [3]*Imperial Cancer Research Fund Histopathology Unit, Lincoln's Inn Fields, London WC2A 3PN, UK.*

The age-incidence curve for testicular cancer has a distinctive shape: it rises to a peak at around age 30, declines to a low level by age 50, and finally increases again in old age. This pattern is also evident in mortality statistics and the rise at older ages is much more marked (Figure 1). Germ cell tumours predominate at younger ages, but it is well known to pathologists and clinicians who treat testicular cancer that the majority of cases occurring at older ages are lymphomas and other non-germ cell tumours arising in the testis (see Peckham, 1981). This fact has been noted in the epidemiological literature (see e.g., Schottenfeld *et al.*, 1980), but the aggregate data (all histologies) covering an age range extending into the older age groups are nevertheless frequently used for epidemiological research purposes without specific attention being drawn to the problem that such data reflect a mixture of tumour types (see e.g., Cancer Research Campaign, 1981; Davies, 1981; Gardner *et al.*, 1983; McDowall & Balarajan, 1986; and most recently Osterlind, 1986). It is likely that there are different causes of at least the major different types of tumour arising in the testis; the use of combined data on tumours with such different histologies can do nothing but hinder our search for clues to their causes, and in particular to the causes of the continuing substantial increase in incidence of seminomas and teratomas of the testis.

The purposes of this note are: (1) to draw the attention of epidemiologists to the fact that epidemiological aspects of germ cell tumours can nevertheless be very satisfactorily studied using aggregate incidence data if attention is restricted to the age range 15 to 50; (2) to draw the attention of the Office of Population Censuses and Surveys (OPCS) and the regional cancer registries to the need to publish all future incidence data by histology (at least broken down into 'germ cell' tumours and 'other'); and (3) to draw the attention of OPCS to the strong possibility that many lymphomas of the testis are being wrongly coded to testis (rather than lymphoma) in current mortality statistics.

**Figure 1** Testicular cancer incidence and mortality rates, England and Wales: 1968–72.

Correspondence: M.C. Pike.
Received 11 December 1986; and in revised form, 31 March 1987.

The reason that many epidemiologists have used aggregate data is, of course, because of the lack of routinely published data by histology. Thus, although the cancer registries of England and Wales code testicular tumours according to site (i.e. testis) and histology (World Health Organisation, 1976), the routinely published cancer incidence statistics for England and Wales (see, e.g., OPCS, 1985*a*) tabulate numbers of registrations by site alone. Death certificates are only coded by site (World Health Organisation, 1978), but lymphomas are allocated a separate code (or codes), so that contrary to the situation with incidence data, a death from a lymphoma arising in the testis is *not* meant to be coded to testis. Cancer mortality statistics (see e.g., OPCS, 1985*b*) are thus only published by site. Such published data (Office of Population Censuses and Surveys, 1970–74; 1979–85; 1984; Registrar General, 1975) were used to calculate the rates shown in Figure 1.

The wide disparity between the mortality and incidence data even in 1968–72 (Figure 1) before the use of sophisticated staging techniques and combination chemotherapy shows that mortality data are unlikely to be useful for epidemiological research purposes. We have, therefore, in this paper concentrated on incidence data.

As we noted above, cancer registries do code testicular tumours by histology (World Health Organisation, 1976), and these incidence data, together with appropriate populations, were kindly supplied to us by the Thames Cancer Registry for the South Thames Region for the 15-year period 1968–82. One thousand seven hundred and seventy new cases of testicular cancer in residents of the South Thames Region were registered during this period. Figure 2a shows the incidence rates by age for all testicular tumours with the characteristic peak around age 30 and a secondary increase after age 65. In Figure 2b the incidence rates shown in Figure 2a are shown divided into seminoma (pure seminoma), teratoma (with or without seminomatous elements) and 'other'. It is clear from this figure that the secondary increase in incidence after age 65 occurs almost entirely in the 'other' category of tumours. Seminomas and teratomas, after the young adult peak, decrease in incidence until age 70–74 with possibly a very minor rise at age 75+. In contrast the incidence of 'other' tumours is low until ages 50–54 but rises steeply thereafter.

We attempted to review the pathology of all the testicular tumours diagnosed in men aged 70 or over, and all diagnoses other than seminoma or teratoma in men under age 70, registered in the South Thames Region in the period 1968–82. We requested material from 89 cases, but, in spite of reminder letters and telephone calls, satisfactory blocks were received for only 24 of them. Immunohistology was carried out on these 24 blocks using a standard indirect immunoperoxidase method. The panel of 5 monoclonal antibodies used comprised PD7/26 (Dako), Cam 5.2 (ICRF), 8B6 (ICRF), anti-desmin (Dako) and anti-vimetin (Dako). PD7/26, CAM 5.2 and 8B6 distinguish between lymphoma, teratoma (with and without seminoma) and pure seminoma (Warnke *et al.*, 1983; Makin *et al.*, 1984). Anti-desmin and anti-vimetin identify sarcomas and distinguish between those

**Figure 2** (a) Testicular cancer incidence rates, South Thames Region: 1968–82; (b) Testicular cancer incidence rates (by histology), South Thames Region: 1968–82.

with and without muscle differentiation (Gabbiani *et al.*, 1981).

Six blocks were received from cases under age 70, all registered as 'carcinoma'. Four of these cases had been reported by the hospital pathologist as teratomas: the diagnoses being wrongly recorded by the Registry. One of the other two cases had been diagnosed as an anaplastic carcinoma; no hospital pathology report was available for the sixth case. On review we diagnosed 5 germ-cell tumours: 1 seminoma (hospital pathologist diagnosis: teratoma), 3 teratomas (hospital pathologist diagnoses: 2 teratomas, 1 anaplastic carcinoma) and 1 yolk sac tumour (hospital pathologist diagnosis: teratoma), so our histological findings were not consistent with the original reports in 3 cases. The sixth block showed a benign adrenal tumour consistent with an origin in ectopic adrenal tissue present in the testis.

There were 18 blocks from patients aged 70 and over; the histological diagnosis recorded by the Registry agreed with the diagnosis made by the hospital pathologist in 17 of the 18 cases (1 hospital pathologist's diagnosis of teratoma was recorded as a seminoma). On review, one of the two hospital pathologists' diagnoses of seminoma was identified as a lymphoma, as was one of the two diagnoses of teratoma, and one of the 11 diagnoses of lymphoma was identified as a seminoma. Our review agreed with the hospital pathologists other 2 diagnoses of germ cell tumours (1 teratoma, 1 yolk sac tumour).

The data for England and Wales shown in Figure 1 show that there is a minor peak in the youngest age groups, 0–4 and 5–9 years. A large proportion of testicular tumours in these age groups are well known to be of two very specific types, paratesticular embryonal sarcomas and yolk sac tumours (Peckham, 1981): although the latter are germ cell tumours, they are distinct from seminomas and the usual teratomas.

Inspection of Figure 2b shows that between the ages of 15 and 50 the overwhelming majority of testicular cancer cases are either seminomas or teratomas, and our pathology review of 'other' tumours in this age range, although admittedly of only a very small number, suggests that a clear majority of these 'other' tumours are also likely to be either seminomas or teratomas. In the absence of data by particular histology, use of aggregate incidence data for the age group 15–49 years can therefore be safely used to investigate the most important issue of the continuing rise in seminomas and teratomas. This procedure is much to be preferred to the current common practice of using data covering all age groups.

We have investigated the change in incidence of germ cell testicular cancer in England and Wales over the three 5 year periods 1968–72, 1973–77 and 1978–82 using this scheme. There was a steady rise in incidence at all ages between 15 and 50; for 1978–82 the cumulative incidence between ages 15 and 50 was 206.1 per 100,000, i.e. 1 in 485 men would be affected between ages 15 and 50, compared to 1 in 625 men in 1968–72 (a 29% rise). [This 29% increase in incidence of testicular cancer between 1968–72 and 1978–82 is likely to be an underestimate, in part due to late registration of cases after the national data are published (Swerdlow, 1986).]

The above analysis shows that national incidence statistics need to be improved by subdividing testicular cancer reporting into at least two categories (germ cell tumours and 'other'). Subdivision of germ cell tumours into seminomas and teratomas is considered by certain investigators to be epidemiologically interesting: it is clear, for example, that their age distributions are different (Figure 2b). However, the 'teratoma' classification includes tumours with seminomatous elements, and we saw above in our limited pathology review that at younger ages our diagnosis disagreed with that of the hospital pathologist in 3 out of 5 cases, and at older ages 2 out of 4 hospital pathologists' diagnoses of germ cell tumours were found to be lymphomas. In these circumstances it is not clear how useful a tumour classification based on reports by multiple hospital pathologists would be. We have, moreover, not found any interesting differences between seminomas and teratomas in our epidemiological studies (Depue *et al.*, 1983; Pike *et al.*, 1986).

Figure 1 shows that there is a marked rise in the mortality rate at older ages and the analysis shown in Figure 2b makes it evident that this rise must be due to 'other' tumours (almost all of which are lymphomas). It is therefore almost certain that a substantial number of deaths from testicular lymphomas are being coded incorrectly to testis: this issue needs to be investigated by the Office of Population Censuses and Surveys.

We thank Helen Jones (Thames Cancer Registry) for supplying incidence data, all the pathologists who kindly supplied blocks and slides, Prof Alan Horwich for helpful comments on an earlier draft of the paper, Eileen Williams and Cynthia Taylor for data processing, and Sybil Farrell and Sarah Jones who prepared the manuscript.

### References

CANCER RESEARCH CAMPAIGN. (1981). Trends in cancer survival in Great Britain. Cancer Research Campaign: London.

DAVIES, J.M. (1981). Testicular cancer in England and Wales: some epidemiological aspects. *Lancet*, **i**, 928.

DEPUE, R.H., PIKE, M.C. & HENDERSON, B.E. (1983). Estrogen exposure during gestation and risk of testicular cancer. *J. Natl Cancer Inst.*, **71**, 1151.

GABBIANI, G., KAPANCI, Y., BARAZZONE, P. & FRANKE, W. (1981). Immunochemical identification of intermediate size filaments in human neoplastic cells. *Amer. J. Pathol.*, **104**, 206.

GARDNER, M.J., WINTER, P.D., TAYLOR, C.P. & ACHESON, A.D. (1983). *Atlas of Cancer Mortality in England and Wales, 1968–78*. John Wiley & Sons: Chichester.

MAKIN, C.A., BOBROW, L.G. & BODMER, W.F. (1984). Monoclonal antibody to cytokeratin for use in routine histopathology. *J. Clin. Pathol.*, **37**, 975.

McDOWALL, M.E. & BALARAJAN, R. (1986). Testicular cancer mortality in England and Wales 1971–80: variations by occupation. *J. Epidemiol. Comm. Hlth*, **40**, 26.

OFFICE OF POPULATION CENSUSES AND SURVEYS. (1970–74). *Registrar General's Statistical Review of England & Wales, 1968–72, Part I Tables, Medical*. Her Majesty's Stationery Office: London.

OFFICE OF POPULATION CENSUSES AND SURVEYS. (1979–85). *Cancer Statistics, Registrations. Series MB1 Nos. 1–14*. Her Majesty's Stationery Office: London.

OFFICE OF POPULATION CENSUSES AND SURVEYS. (1984). *OPCS Monitor. PP1 84/1*. Office of Population Censuses and Surveys: London.

OFFICE OF POPULATION CENSUSES AND SURVEYS. (1985*a*). *Cancer Statistics, Registrations 1982. Series MB1 No. 14*. Her Majesty's Stationery Office: London.

OFFICE OF POPULATION CENSUSES AND SURVEYS. (1985*b*). *Mortality Statistics 1984*. Her Majesty's Stationery Office: London.

OSTERLIND, A. (1986). Diverging trends in incidence and mortality of testicular cancer in Denmark, 1943–82. *Br. J. Cancer*, **53**, 501.

PECKHAM, M.J. (ed.) (1981). *The Management of Testicular Tumours*. Edward Arnold: London.

PIKE, M.C., CHILVERS, C. & PECKHAM, M.J. (1986). Effect of age at orchidopexy on risk of testicular cancer. *Lancet*, **i**, 1246.

REGISTRAR GENERAL. (1975). *Registrar General's Statistical Review, Supplement on Cancer, 1968–70*. Her Majesty's Stationery Office: London.

SCHOTTENFELD, S., WARSHAUER, M.E., SHERLOCK, S. & 3 others (1980). The epidemiology of testicular cancer in young adults. *Am. J. Epidemiol.*, **112**, 232.

SWERDLOW, A.J. (1986). Cancer registration in England and Wales: some aspects relevant to interpretation of the data. *J. Roy. Stat. Soc.*, Series A, **149**, 146.

WARNKE, R.A., GATTER, K.C., FALINI, B. & 7 others (1983). Diagnosis of human lymphoma with monoclonal antileukocyte antibodies. *N. Engl. J. Med.*, **309**, 1275.

WORLD HEALTH ORGANISATION. (1976). International *Classification of Diseases for Oncology*. World Health Organisation: Geneva.

WORLD HEALTH ORGANISATION. (1978). Manual of the *International Statistical Classification of Diseases, Injuries and Causes of Death*. World Health Organisation: Geneva.